# Gender, Socioeconomic, and Health Characteristics Associated with Influenza Vaccination Coverage (VC) among Italian Healthcare Workers: Secondary Analysis of a National Cross-Sectional Survey

**DOI:** 10.3390/healthcare8030298

**Published:** 2020-08-26

**Authors:** Pamela Barbadoro, Aura Brighenti, Giorgia Acquaviva, Alessandro Catalini, Francesca Diotallevi, Alberto Lino Masiero, Vincenzo Montagna, Marcello Mario D’Errico

**Affiliations:** Section of Hygiene, Department of Biomedical Science and Public Health, School of Medicine, Università Politecnica delle Marche, Via Tronto 10/a, 60126 Ancona, Italy; p.barbadoro@staff.univpm.it (P.B.); igiene.marche@gmail.com (A.B.); giorgia.acquaviva11@gmail.com (G.A.); musa@univpm.it (A.C.); francesca.diotallevi92@gmail.com (F.D.); ginosr@gmail.com (A.L.M.); specigiene2019@gmail.com (V.M.)

**Keywords:** influenza, healthcare workers, vaccine coverage, socioeconomic factors

## Abstract

Influenza epidemics pose a great overload over health-care facilities with an increase in the burden of disease for patients and healthcare costs. Despite a well-established amount of research in the area, vaccination rates show room for improvement and more research is needed in finding systematic interventions useful in improving healthcare workers (HCWs) vaccination coverage (VC). The purpose of this study was to describe the self-reported frequency of influenza immunization in HCWs and to identify demographic, socioeconomic, lifestyle, and health factors associated with this practice in Italy. Data about 5823 workers participating in the Italian national survey about health and healthcare services utilization are analyzed in the present study. Overall, 18.8% of HCWs reported being vaccinated against seasonal flu. In the multilevel regression, older workers had a higher likelihood of vaccine uptake (OR: 6.07; 95% CI 4.72–7.79), similar to those with chronic conditions or poor self-perceived health status (OR: 2.18 95% CI 1.17–4.09). On the other hand, the results highlighted a lower rate of VC in female HCWs (OR: 0.73 95% CI 0.61–0.86). Data confirm the low compliance towards flu immunization among Italian HCWs and highlight an important gap to be investigated in women.

## 1. Introduction

Influenza epidemics pose a great overloading of healthcare facilities with an increase in the burden of disease for patients and costs for the healthcare systems worldwide [1,2]. Besides being at increased risk of contracting flu, healthcare workers (HCWs) may also play a part in the spread of infections in the hospital settings [3]. Therefore, vaccinations are the most important measures for infection control and are aimed not only at protecting workers but above all, to avoid flu transmission to vulnerable patients, with considerable benefits, especially in at-risk groups [4,5,6,7,8,9]. For this reason, health authorities have made recommendations concerning vaccination. In accordance to the World Health Organization strategy the current European and Italian National Vaccine Prevention Plan 2017–2019 recommend anti-influenza vaccination for HCWs [10,11]. Furthermore, anti-influenza immunization strategies, following international guidelines, target subjects aged ≥65 years and those at risk of complications. Despite the wide evidence that the HCWs’ vaccinations contribute to promoting safety, in many developed countries including Italy, vaccination coverage (VC) is low and still shows room for improvement [12]. Similar evidence has been gathered in an Italian setting [13,14,15,16]. However, recent shreds of evidence relating to the Italian context have shown that specific organizational and educational interventions may have mixed results in ameliorating influenza VC [17,18,19,20]. Thus, research is important in finding variables useful in designing systematic interventions aimed at implementing HCWs vaccination coverage. The purpose of this study is to describe the self-reported frequency of influenza immunization in HCWs and to identify socioeconomic, lifestyle and health factors associated with this practice in HCWs living in Italy.

## 2. Methods

This is a secondary analysis of data derived from the multipurpose Italian Health Interview Survey about health and healthcare use carried out by the Italian National Institute of Statistics (ISTAT). Each participating subject completed a self-administered questionnaire and had a face-to-face interview with ISTAT data collectors. The latest edition of this survey, completed between 2012 and 2013, gathered data on 119,073 individuals, representative of the Italian general population living in households.

In the present analysis, socio-demographic and health data of the 5823 workers employed in the health care sector were examined. In particular, the following variables were included: age class (less than 44 years old; between 45–64 years old; more than 65 years old), area of residence (northeast, northwest, central, south and the islands); marital status was classified into single, married/cohabiting, separated/divorced or widowed; educational level was classified into post-graduate, graduate, undergraduate, or high school or lower education. The survey also provided lifestyle information, perceived health status (excellent; very good; good; fair and poor), and the presence of chronic diseases, in general (including severe asthma), with particular attention for cardiovascular diseases (hypertension, angina, and acute events like heart attack and stroke) and diabetes.

Bivariate analyses were performed to study the association of vaccine influenza uptake with relevant variables using Chi-square tests, as appropriate. Mixed effect logistic regression models have been built to adjust for confounders and to evaluate the factors independently associated with vaccination (1, if vaccinated; 0 if not vaccinated), accounting for the cluster sampling design, at the regional level of the survey. The significance level for variables to enter the multiple logistic regression models was set at <0.2 and for removing them from the model the level was set at >0.4. The level of significance was set at 0.05. Analyses have been performed with STATA, version 15 (Stata Corp.).

## 3. Results

Among the selected sample, the overall rate of HCWs declaring to have received an influenza vaccination in the past 12 months was 18.08% (95% confidence interval, CI 17.09–19.07). Bivariate analysis (Table 1) has highlighted higher immunization rates in males (22.83%, n = 1818) and older workers, with proportions reaching 48.26% (n = 1005) in HCWs over 65. Education level showed a mixed trend, with higher levels in post-graduate (doctoral, specialization and master’s degrees) and undergraduate HCWs, with 19.02% (n = 3865) and 19.97% (n = 1297), respectively, versus 8.88% (n = 665) in graduated. High immunization levels were found for those suffering from chronic diseases also, such as cardiovascular diseases (CVD, 34.05%, n = 1448), COPD (43.82%, n = 178) and diabetes (45.28%, n = 318), as well as in obese HCWs (26.00%, n = 623). Another important variable associated with vaccination was self-perceived health status (up to 50.82% in those being in very poor conditions, n = 61). Vaccination coverage (VC) among former smokers showed higher rates (23.41%, n = 1474).

Multilevel regression analysis (Table 2) confirms that older (people who are >64 years old, OR: 6.88 95% CI 5.31–8.90), widowed workers (OR: 1.24 95% CI 0.96–1.60) and those who are affected by chronic diseases such as diabetes (OR: 1.94 95% CI 1.48–2.54), COPD (OR: 1.26 95% CI 1.06–1.51), cardiovascular (OR 1.54, 95%CI, 1.30–1.83), or other diseases (OR: 1.56 95% CI 1.20–2.01) had a higher likelihood of being vaccinated. Likewise, HCWs reporting low or very low self-perceived health status have higher odds of vaccination (OR: 1.59 95% CI 1.08–2.32 and OR: 2.18 95% CI 1.17–4.09). On the other hand, multilevel regression confirms that females usually report a lower rate of VC (OR: 0.73 95% CI 0.61–0.86).

## 4. Discussion

Despite continuous support, Italian HCWs confirm their low compliance with the national and international recommendations for influenza immunization. The mean coverage stops at 18.08% (95% CI 17.09–19.07), a value that is lower than that registered in the previous edition of the survey (20.8%, 95% CI 19.7–21.9) [16], with rates as low as 7.04% in the younger age group. This result is in agreement with previous studies revealing that knowledge of recommended vaccinations, as well as coverage, may be insufficient in Italian HCWs [13,14,15,16], thus representing a risk for healthcare safety and outbreaks surge [21,22]. Results are in line with previous studies, where lower VC has been associated with limited awareness of the cost-effectiveness (CE) profile of influenza vaccination in adults, as HCWs tend to have a better knowledge of recommendations for higher risk groups [23]. To improve vaccine compliance, the international Public Health organization recommend on-site influenza vaccination as a proven and cost-effective strategy that increases productivity, reduces overall absenteeism, and prevents direct health-care cost [24]. Dealing with the educational level, the trend toward a lower VC in nurses and other professionals previously seen in an Italian sample [16], was confirmed in the present survey, with undergraduates declaring VC similar to that of post-graduates but graduated (including most Italian nurses) with lower levels of VC. However, multilevel modeling has reduced the relative importance of educational level found in the bivariate analysis in this survey, thus confirming the complex interplay between education and vaccine uptake. This result is in line with previous empirical studies on vaccination acceptance providing mixed evidence of the effect of educational level on influenza vaccine uptake. A review of the literature discussing cross-country comparisons has shown that the effect of education for influenza VC is not constant, supporting the idea that educational differences in vaccination uptake are contextual upon the reflexivity of the society in which the respondent lives. In this context, more educated people living in more reflexive modernized countries tend to oppose vaccination against seasonal flu more than those highly educated living in less advanced societies [25]. This complex interplay between influenza VC and educational level has been addressed also by a review of the literature specifically analyzing the association between Health Literacy and vaccination and concluding that the link remains unclear [26]. Among the various variables associated to VC, we should discuss the role of gender found in this analysis, as well as in the previous edition of a similar survey [16], and in a recent study from Northern Italy, where the male sex was associated to a significantly lower prevalence ratio of never being vaccinated before, thus underlining a possible hesitancy in women [20]. Nevertheless, similar results relating to the lower coverage have recently been highlighted in older women in the USA [27]. Gender inequality is a well-known gap in immunization rate, especially in childhood, but these results highlight a potential life-time gap to be investigated. Holistic/systemic approaches are necessary to unravel the complexity of the interaction between gender, socioeconomic, environmental/community factors on immunization, to target specific implementation strategies that may operate differently within and across gender and other variables [28]. The important role of the healthcare organization has been highlighted by the intraclass-correlation of results showing a significant variation in VC through the regions, now quite autonomous in Italy, in the framework of a federalist reform of the Italian healthcare system and highlight the importance of the implementation strategies in achieving VC.

A general limitation of the study may be linked to the lack of a detailed description of individuals’ degrees within the healthcare organizations, as already discussed [16].

## 5. Conclusions

Self-reported vaccine coverage among Italian HCWs still shows room for improvement. HCWs are pivotal in counseling patients, as well as mothers, regarding immunization, therefore they should be more aware of the prevention of infectious disease through active immunization [29]. Again, the reduced VC found in women is alarming, and maybe even more important, given their influence on child care. Gender-related barriers should be considered in HCW vaccine coverage analysis, and addressed as a critical, cross-cutting, and influencing factor in immunization programs [30] as well as in the design of specific educational programs targeting the issue of low vaccine uptake.

## Figures and Tables

**Table 1:** Vaccine coverage of healthcare workers and their children during the previous 12 months, distribution of socioeconomic, lifestyle, and health characteristics of professionals.

Variables		N	% Vaccinated	*p* ^a^
Sex	Male	1818	22.83	
	Female	4005	15.93	*
Age Group	<44	2088	7.04	
	45–64	2730	15.42	
	65 or more	1005	48.26	*
Marital Status	Single	3283	19.07	
	Married/Cohabiting	1380	10.65	
	Separated/Divorced	751	14.91	
	Widowed	409	41.08	*
Education	Post-Graduate	3865	19.02	*
	Graduate	665	8.88	
	Undergraduate	1297	19.97	
CVD	No	4375	12.80	*
	Yes	1448	34.05	
COPD	No	5645	17.27	*
	Yes	178	43.82	
Diabetes	No	5505	16.51	*
	Yes	318	45.28	
Other Chronic Diseases	No	4955	13.95	*
	Yes	868	41.71	
BMI	Underweight	207	13.53	*
	Healthy Weight	3278	15.10	
	Overweight	1715	21.46	
	Obese	623	26.00	
Smoking Habit	Smoker	1212	14.69	*
	Former Smoker	1474	23.41	
	Never Smoked	3137	16.90	
Self-Perceived Health Status	Very Good	937	10.25	
	Good	3158	14.72	*
	Fair	1402	25.53	
	Low	265	38.87	
	Very Low	61	50.82	
Medical Examination during the Previous 4 Weeks	No	3883	15.31	*
	Yes	1937	23.64	

* *p* ^a^ < 0.05, Chi-square test.

**Table 2:** Variables associated with HCWS’s vaccine coverage during the previous 12 months results of multilevel modeling.

Variables		OR	CI 95%	*p* ^a^
Sex	Male	1		
	Female	0.73	0.61–0.86	*
Age Group	<44	1		
	45-64	1.86	1.49–2.32	*
	65	6.88	5.31–8.90	*
Marital Status	Single	1		
	Married/Cohabiting	0.86	0.69–1.07	
	Separated/Divorced	0.88	0.70–1.11	
	Widowed	1.24	0.96–1.60	
Education	Post-Graduate	1		
	Graduate	0.87	0.63–1.20	
	Undergraduate	1.42	0.66–3.02	
CVD	No	1		
	Yes	1.54	1.30–1.83	*
COPD	No	1		
	Yes	1.26	1.06–1.51	*
Diabetes	No	1		
	Yes	1.94	1.48–2.54	*
Other Chronic Disease	No	1		
	Yes	1.56	1.20–2.01	*
BMI	Underweight	1		
	Healthy Weight	0.86	0.55–1.35	
	Overweight	0.88	0.55–1.39	
	Obese	0.96	0.59–1.56	
Smoking Habit	Never Smoked	1		
	Former Smoker	1.15	0.92–1.43	
	Smoker	1.11	0.91–1.36	
Self-Perceived Health Status	Very Good	1		
	Good	1.06	0.83–1.36	
	Fair	1.19	0.90–1.57	
	Low	1.59	1.08–2.32	*
	Very Low	2.18	1.17–4.09	*
Medical Exam during the Previous 4 Weeks	No	1		
	yes	1.16	0.99–1.36	

Intraclass correlation coefficient, ICC 16.9% (95% CI 8.60–33.30); * *p* ^a^ < 0.05.
